# Integrative 5-Methylcytosine Modification Immunologically Reprograms Tumor Microenvironment Characterizations and Phenotypes of Clear Cell Renal Cell Carcinoma

**DOI:** 10.3389/fcell.2021.772436

**Published:** 2021-12-08

**Authors:** Wenhao Xu, Wenkai Zhu, Xi Tian, Wangrui Liu, Yuanyuan Wu, Aihetaimujiang Anwaier, Jiaqi Su, Shiyin Wei, Yuanyuan Qu, Hailiang Zhang, Dingwei Ye

**Affiliations:** ^1^ Department of Urology, Fudan University Shanghai Cancer Center, Shanghai, China; ^2^ Department of Oncology, Shanghai Medical College, Fudan University, Shanghai, China; ^3^ Department of Neurosurgery, Affiliated Hospital of Youjiang Medical University for Nationalities, Baise, China; ^4^ Department of Gastroenterology, Naval Medical Center of PLA, Naval Military Medical University, Shanghai, China

**Keywords:** clear cell renal cell carcinoma, 5-methylcytosine, tumor microenvironment, renal cell carcinoma (RCC) clear cell renal cell carcinoma (CCRCC), immune checkpoint therapies, prognosis, machine learning algorithm

## Abstract

The tumor microenvironment (TME) affects the biologic malignancy of clear cell renal cell carcinoma (ccRCC). The influence of the 5-methylcytosine (m^5^C) epigenetic modification on the TME is unknown. We comprehensively assessed m^5^C modification patterns of 860 ccRCC samples (training, testing, and real-world validation cohorts) based on 17 m^5^C regulators and systematically integrated the modification patterns with TME cell-infiltrating characterizations. Our results identified distinct m^5^C modification clusters with gradual levels of immune cell infiltration. The distinct m^5^C modification patterns differ in clinicopathological features, genetic heterogeneity, patient prognosis, and treatment responses of ccRCC. An elevated m^5^C score, characterized by malignant biologic processes of tumor cells and suppression of immunity response, implies an immune-desert TME phenotype and is associated with dismal prognosis of ccRCC. Activation of exhausted T cells and effective immune infiltration were observed in the low m^5^C score cluster, reflecting a noninflamed and immune-excluded TME phenotype with favorable survival and better responses to immunotherapy. Together, these findings provide insights into the regulation mechanisms of DNA m^5^C methylation modification patterns on the tumor immune microenvironment. Comprehensive assessment of tumor m^5^C modification patterns may enhance our understanding of TME cell-infiltrating characterizations and help establish precision immunotherapy strategies for individual ccRCC patients.

## Introduction

Renal cell carcinoma (RCC) is the most common malignancy of the urinary system, accounting for approximately 3.8% of all newly diagnosed cancers. The incidence of RCC is increasing by 1.1% each year (Siegel et al., 2020). Clear cell RCC (ccRCC), which originates from proximal tubule epithelial cells, is the most common histology type of RCC, accounting for approximately 80% of all RCC cases (Capitanio and Montorsi, 2016; Linehan and Ricketts, 2019). The Von Hippel–Lindau (VHL) gene is frequently mutated in ccRCC, and mutations in *BAP1*, *PBRM1*, *SETD2*, and *PIK3CA* are also commonly observed in ccRCC. Studies show that mutations in these genes influence the prognosis and treatment response of ccRCC patients (Clark et al., 2019; Linehan and Ricketts, 2019). The standard first-line treatment strategy for metastatic or advanced ccRCC mainly involves tyrosine kinase inhibitors, such as sunitinib and sorafenib, that target vascular endothelial growth factor receptors. Over the past few decades, rapid progress has been made in immunotherapy as a new treatment strategy for cancer (Xu et al., 2019; Braun et al., 2020; Motzer et al., 2020).

DNA methylation is one of the most researched epigenetic modifications and is linked to the development of human malignancies (Qian et al., 2020). The main type of DNA methylation is the presence of an additional methyl group on the 5 position of cytosine (5-methylcytosine, m^5^C) (Choi et al., 2021). The m^5^C modification was the first discovered epigenetic marker and plays an important role in regulating the transcriptome profiles and carcinogenesis process of solid tumors, which often harbor aberrant DNA methylation (Martisova et al., 2021). The m^5^C modification is frequently found in large clusters called CpG islands, which are present in gene-promoter regions and suppress gene transcription (Chen et al., 2019; Palei et al., 2020). A series of enzymes, called writers, readers, and erasers, is responsible for adding, recognizing, and removing the m^5^C modifications, respectively (Rausch et al., 2020). Some tumor-suppressor genes are silenced as a consequence of hypermethylation in the promoter regions. Therefore, DNA methylation represents a potential signature and promising treatment target for human malignancies. Investigation of m^5^C epigenetic modifications and their regulation of gene expression may, thus, provide insights into the mechanisms underlying cancer development.

The 5-methylcytidine modification occurs on both DNA and RNA. The major epigenetic mark in mammalian DNA is m^5^C, which is associated with carcinogenesis and tumorigenesis of various cancers (Greenberg and Bourc’his, 2019). The phenotype of tumor microenvironment (TME) is dynamically regulated by cell signaling transduction and epigenetic drivers, which are critical factors influencing the efficacy of immunotherapy and both extrinsic and intrinsic resistance pathways. DNA methyltransferase enzymes (DNMTs) methylate CpG islands in gene promoters, and aberrant expression or activity of DNMTs can lead to tumorigenesis and aggressive progression (Zhang et al., 2018). Additionally, upregulated DNMT1 is shown to be necessary for maintaining cancer stemness and is associated with poor clinical outcome of cancers. DNMT1 is also shown to regulate the inhibitory function of Foxp3^+^ T-regulated cells (Piperi et al., 2008; Wang et al., 2013; Zagorac et al., 2016). Therefore, comprehensively exploring the biological activities of epigenetic drivers in tumor phenotypes and TME is important (Xu et al., 2021a).

In this study, we examine the potential influence of DNA m^5^C regulators on the clinical malignant characteristics and TME of ccRCC. We first constructed m^5^C clusters using large-scale samples and algorithms and evaluated the relationship of m^5^C clusters with immune cell infiltration, the DNA variation landscape, and immunotherapy in ccRCC.

## Materials and Methods

### Sample Collection and Data Preprocessing

Gene expression, copy number variants, tumor somatic mutations, and matched clinical information of ccRCC from The Cancer Genome Atlas (TCGA) cohort were obtained. Gene expression data of 93 ccRCC tumors from the Clinical Proteomic Tumor Analysis Consortium (CPTAC) were obtained at https://proteomics.cancer.gov/programs/cptac. In addition, RNA-seq and clinical data of 91 ccRCC patients from the RECA-EU cohort were available from the International Cancer Genome Consortium (ICGC, https://dcc.icgc.org/) database and included in this study. Patients without overall survival information were removed from further analysis. In addition, 232 ccRCC samples with proteomics sequencing data with available clinical and pathologic electronic records were enrolled from our institute, Fudan University Shanghai Cancer Center (FUSCC, Shanghai, China). In total, 860 ccRCC tumor samples were included for analysis. Batch effects from nonbiological technical biases were corrected using the “ComBat” algorithm of sva package and the fragments per kilobase of transcript per million values were transformed into transcripts per kilobase million values.

### Unsupervised Clustering for 17 m^5^C Regulators

A total of 17 m^5^C regulators were extracted from the integrated gene expression profiles to identify different m^5^C modification patterns. The 17 m^5^C regulators included three writers (*DNMT1*, *DNMT3A*, and *DNMT3B*), three erasers (*TET1*, *TET2*, and *TET3*), one regulating factor (*DNMT3L*), and 10 readers (*MECP2*, *MBD1*, *MBD2*, *MBD3*, *MBD4*, *UHRF1*, *UHRF2*, *ZBTB4*, *ZBTB38*, and *ZFP57*). The “ConsensusClusterPlus” R package was used to classify patients for further analysis, and 1000 times repetitions were conducted to ensure the stability of the classification (Wilkerson and Hayes, 2010). Overall survival was compared between patients with different m^5^C modification patterns.

### Gene Set Variation Analysis (GSVA) and TME Cell Infiltration Estimation

GSVA, a commonly employed method for estimating the variation in pathways (Hänzelmann et al., 2013), was used to evaluate the potential biological differences between the m^5^C modification patterns using the “GSVA” R package. To estimate the TME cell infiltration, we applied the single-sample gene set enrichment analysis algorithm to evaluate the relative abundance of immune cells in the ccRCC TME. The reference gene sets for quantifying the immune cells were obtained from a previous study (Charoentong et al., 2017), and the examined immune cells included mast cells, monocyte, macrophage, activated CD4^+^ T cells, and other types of immune cells. Immune cell abundance was compared between m^5^C modification patterns, and the prognostic significance of each immune cell was also evaluated based on the overall survival information.

### Differential Gene Expression Analysis and Functional Enrichment Analysis

To explore the potential biological differences between m^5^C modification patterns, the limma package was used to identify differentially expressed genes (DEGs), and the threshold value was set as *p* < .05, |logFC|≥3 (Ritchie et al., 2015). Functional enrichment analyses were carried out to explore the potential functions of the DEGs. The expression profiles of DEGs were extracted, and unsupervised clustering was applied again to identify the subgroups; the subgroups were defined as m^5^C gene clusters.

### Identifying m^5^C Score as the m^5^C Gene Signature

A scoring system was constructed to evaluate the m^5^C modification patterns, and we termed it as m^5^C score. Univariate Cox regression was used to evaluate the prognostic value for each gene, and the genes with prognostic significance were extracted for further analysis. Random forest analysis and principal component analysis were used to construct the m^5^C relevant gene signature. Both principal components 1 and 2 were enrolled to calculate the signature scores, and the m^5^C score was defined as follows: ${\text{m}}^{5}\text{C}\cdot \text{score }=\;\sum \left({\text{PC}2}_{\text{i}}{+\text{PC}2}_{\text{i}}\right)$ .

### Copy Number Variant Analysis, Immunotherapy Response Prediction, and IC50 Evaluation

To explore potential associations between copy number variants and m^5^C score, Genomic Identification of Significant Targets in Cancer (version 2.0) was used to identify significantly amplified or deleted regions using TCGA copy number data (Beroukhim et al., 2007; Mermel et al., 2011). Q ≤ 0.05 was defined as significant, and the confidence interval was set to 0.95. Tumor immune dysfunction and exclusion (TIDE) was used to estimate the immunotherapy response based on the expression profiles (Jiang et al., 2018). Thus, the associations between m^5^C score and immunotherapy response were evaluated. The pRRophetic package was used to predict the half-maximal inhibitory concentration (IC50) of chemotherapy drugs in the high and low m^5^C score groups.

### Immunohistochemistry (IHC)

IHC was performed to evaluate the expression levels of Ki-67 (ab15580; Abcam), CD4 (RMA-0620, Maxim, China), CD8 (RMA-0514, Maxim, China), Glut-1 (ab115730; Abcam), PD-L1 (ab205921; Abcam), CXCL13 (ab246518; Abcam), TGF-β (ab189778; Abcam), FASN (ab99359; Abcam), CK (Kit-0009, Maxim, China), and FoxP3 (98,377, CST) following previously described procedures (Xu et al., 2021a; Xu et al., 2021b). Opal multispectral was implemented to identify differential immune cell infiltration and PD-L1 expression in different groups on a multispectral imaging system (Vectra^®^ Polaris™, Shanghai, China).

### Statistical Analysis

A Wilcox test was used to compare differences between two groups. The Kaplan–Meier method was used to conduct survival analysis, and the cutoff value was defined *via* the survminer package. A log-rank test was used to detect the significance. The receiver operating characteristic curve (ROC) was drawn to evaluate the predictive ability for immunotherapy response.

## Results

### The Overall Depiction of Genetic Variation of m^5^C Regulators in ccRCC

**FIGURE 1:**
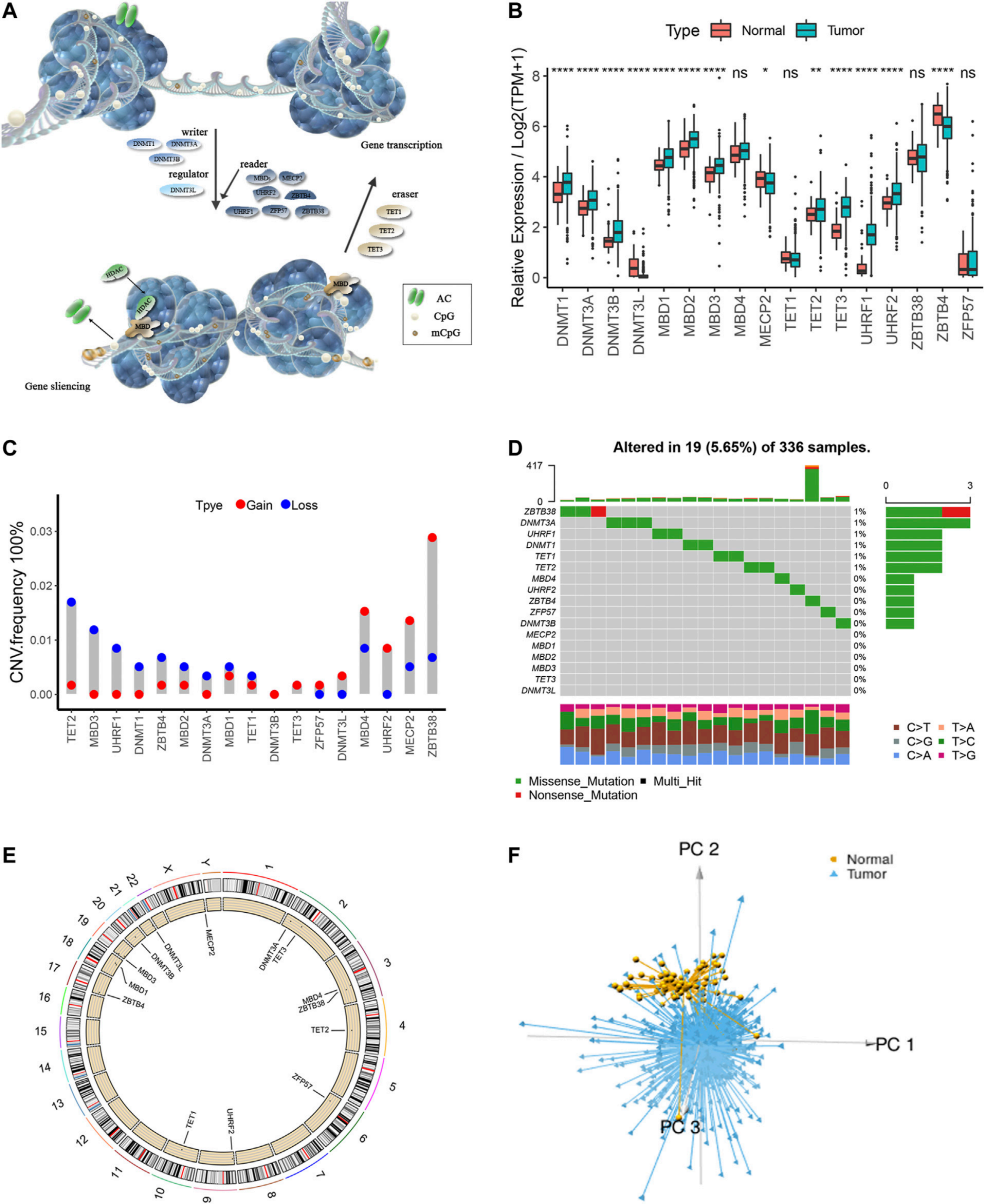
The overall depiction of genetic variations of m^5^C regulators in ccRCC. **(A)** The dynamic reversible process of m^5^C DNA methylation mediated by regulators as well as their potential biological functions for ccRCC are summarized. **(B)** Comparison of the expression levels of 17 m^5^C regulators in 530 ccRCC samples and >12,000 para-cancer tissues. **(C)** Copy number variations (CNVs) of the 17 m^5^C regulators in ccRCC from TCGA cohort. **(D)** Somatic variant landscape of the 17 m^5^C regulators in ccRCC from TCGA cohort. **(E)** The location of CNV alterations of m^5^C regulators on chromosomes. **(F)** Principal component analysis of ccRCC samples from the TCGA cohort based on the expression of the 17 m^5^C regulators.

A total of 17 m^5^C regulators including three writers, three erasers, one regulating factor, and 10 readers were manually identified in this study. The dynamic reversible process of m^5^C DNA methylation mediated by regulators as well as their potential biological functions for ccRCC are summarized in Figure 1A. We detected significant differences in the expressions of m^5^C regulators between ccRCC and para-cancer tissues (*p* < .05) (Figure 1B). Analysis of CNV frequency indicated that CNV alterations were prevalent in the 17 m^5^C regulators, and half of the m^5^C regulators more frequently showed copy number amplification compared with copy number loss (Figure 1C). Besides this, in DNA variation profiles, we found 19 experienced samples of m^5^C regulators with a frequency of 5.65% among 336 ccRCC samples from TCGA (Figure 1D). The location of CNV alterations of the m^5^C regulators on chromosomes is shown in Figure 1E. Notably, ccRCC samples could be distinguished from normal samples completely based on the expression pattern of these m^5^C regulators (Figure 1F). These findings suggest a high degree of m^5^C modification–mediated intertumoral heterogeneity of genetic and expressional alteration landscape between ccRCC and adjacent normal samples, suggesting that the aberrant expression of m^5^C regulators may play an essential role in ccRCC malignancy.

### Machine Learning Algorithms Identify m^5^C Modification Patterns Mediated by the Regulators

**FIGURE 2:**
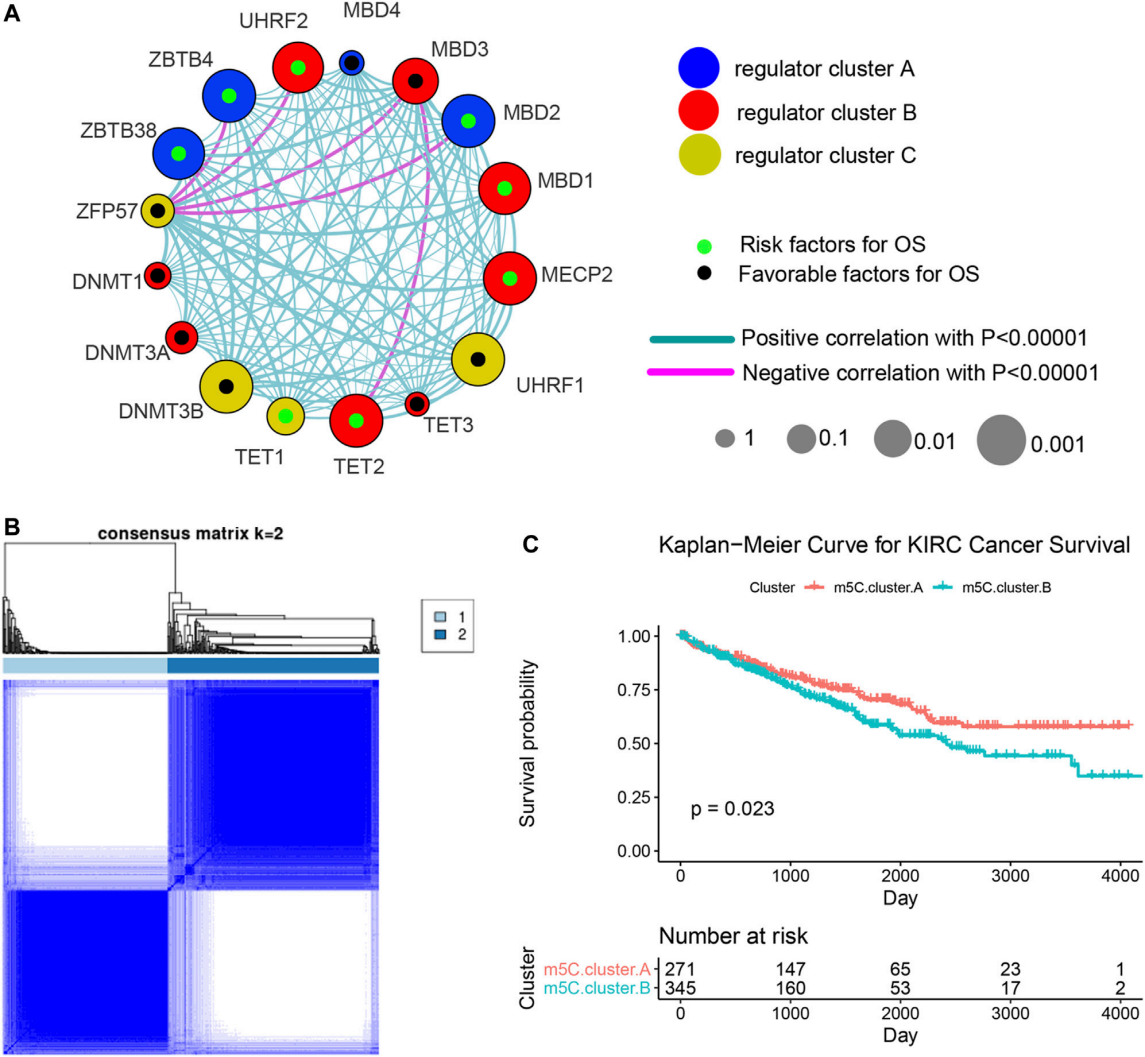
Machine learning algorithms identify m^5^C modification patterns mediated by the regulators. **(A)** Comprehensive landscape of the interactions of the 17 m^5^C regulators, interaction network, and the prognostic implications for ccRCC patients. **(B)** Unsupervised clustering based on expression of 17 m^5^C regulators. **(C)** Overall survival curves of ccRCC patients in the two m^5^C modification pattern clusters.

Three data sets (both proteome and transcriptome) with available survival and clinicopathological information (TCGA, CPTAC, and RECA-EU) were included in one meta-cohort. Figure 2A shows the comprehensive landscape of the interaction of the 17 m^5^C regulators, interaction network, and the prognostic implications for ccRCC patients. The results identified TET2, MBD1, MBD2, MECP2, ZBTB4, ZBTB38, and UHRF2 as protumorigenesis indicators for ccRCC, and MBD3, UHRF1, and DNMT3B were identified as significant favorable factors for ccRCC. We also found that expression of the m^5^C regulators in the same functional category exhibited remarkable correlations, and a marked association was displayed among writers, regulators, erasers, and readers. For instance, whether ccRCC tumors with a high writer gene expression exhibit a high eraser gene expression normally depended on the different writer and eraser genes. However, we found that tumors with high expression of the m^5^C reader gene *ZFP57* showed low expression of some reader genes (*ZBTB4*, *UHRF2*, *MBD3*, and *MBD2*) although the high expression of other reader genes was not affected. These results imply that a cross-talk among the genes encoding m^5^C writers, readers, regulators, and erasers could play essential roles in the malignancy of different m^5^C modification patterns and tumor immune microenvironment characterization among individual ccRCC samples.

We next used the ConsensusClusterPlus R package to identify ccRCC patients with qualitatively different m^5^C modification patterns based on the transcriptional expression of 17 m^5^C regulators. Two distinct modification patterns were classified using unsupervised clustering: m^5^C cluster A (including 271 cases) and m^5^C cluster B (354 cases) (Figure 2B). Survival analysis of patients in the two clusters revealed that patients with the m^5^C cluster A modification pattern showed improved survival compared with patients with the m^5^C cluster B pattern (Figure 2C).

### Evaluation of TME Characterizations and Immune Contexture Proportion in Distinct m^5^C Modification Patterns

**FIGURE 3:**
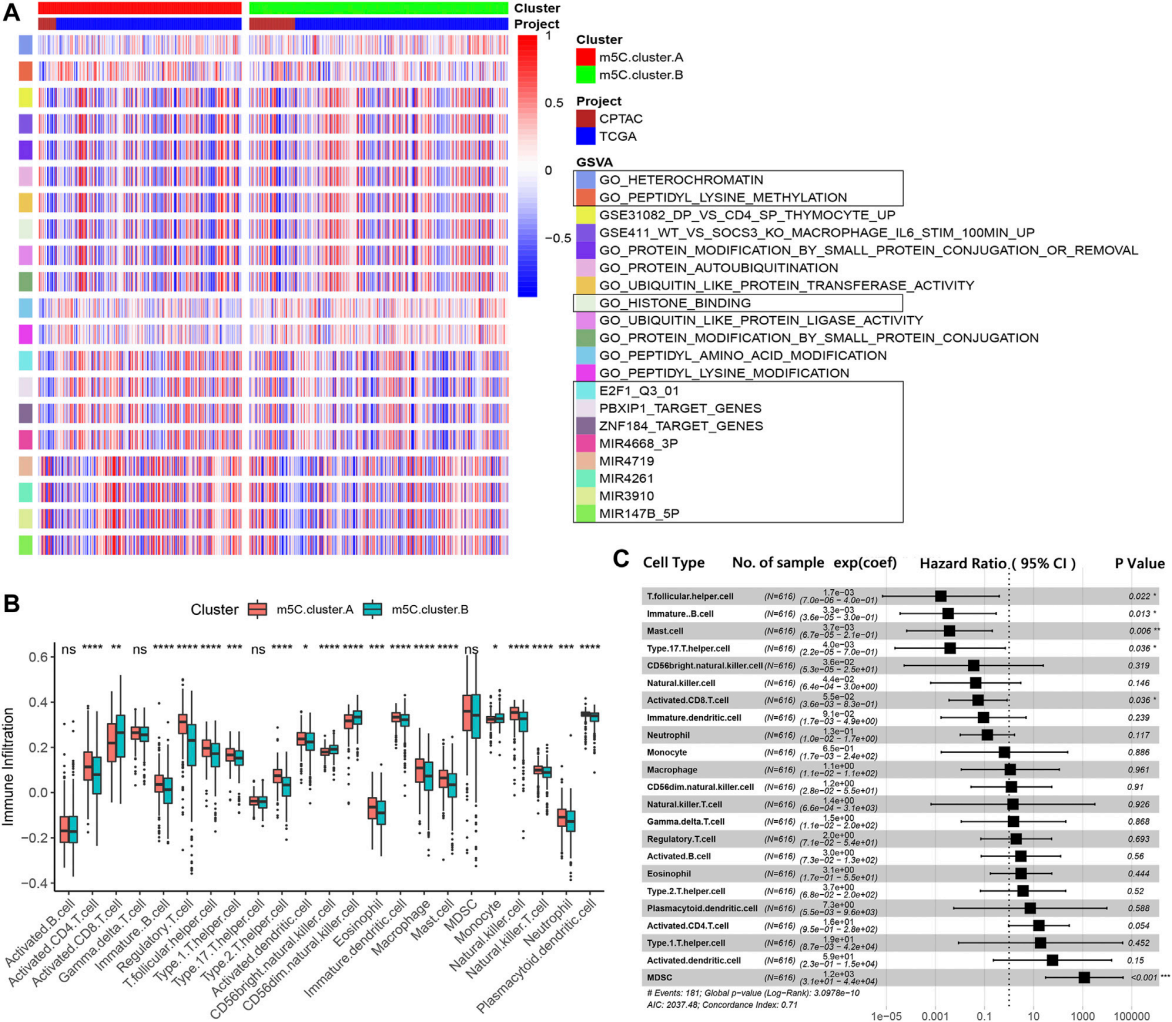
TME characterizations and infiltrating immune cells in the two m^5^C modification patterns. **(A)** GSVA results of the two m^5^C modification patterns. **(B)** Estimation of immune cell infiltration in the two m^5^C modification patterns. **(C)** Univariate regression analysis of various types of immune cells.

To investigate the clinical differences and biological processes between the two distinct m^5^C modification patterns, we constructed a clustering heat map showing differentially expressed m^5^C regulators and clinical information, including age, sex, stage, and survival status in the two m^5^C modification patterns (Supplementary Figure S1). GSVA enrichment analysis indicated that m^5^C clusters mainly differ in heterochromatin, peptidyl modification pathways, and microRNA post-transcriptional regulation (Figure 3A). The ccRCC samples in m^5^C cluster A showed prominent upregulation in E2F1, miR-147B, miR-3910, miR-4261, miR-3689-3p, miR-4719, PBXIP1, and ZNF184 targeted regulation and downregulation in peptidyl modification processes, such as histone binding, peptide amino acid modification, protein autoubiquitination, ubiquitin-like protein ligase, and transferase activities (Supplementary Figure S2A).

We next examined immune cell infiltration to assess differences in the immune context of the TME between m^5^C modification patterns. m^5^C cluster A was remarkably rich in innate immune cell infiltration and the active immune response process with a high abundance of activated CD4 T cells, immature B cells, regulatory T cells, Tfh cells, dendritic cells, eosinophils, macrophages, mast cells, natural killer cells, and neutrophils (Figure 3B). The results from GSVA analyses demonstrate that the m^5^C cluster A modification pattern, which predicts favorable clinical outcome, was significantly associated with antitumor immune responses. Therefore, we hypothesized that the peptidyl modification inactivation in m^5^C cluster A may be involved in the antitumor effects of immune cell infiltration related to this cluster.

We further assessed the prognostic implications of immune cell infiltration in ccRCC (Figure 3C). Univariate Cox analysis indicated that T follicular helper cells (*p* = .022), immature B cells (*p* = .013), mast cells (*p* = .006), type 17 T helper cells (*p* = .036), and activated CD8 T cells (*p* = .036) could serve as independent prognostic protective factors in ccRCC, and MDSC (*p* < .001) was a remarkable risk indicator for 616 ccRCC patients from the TCGA and CPTAC cohorts (Figure 3C). When clinicopathological factors were analyzed, we found no significant differences in the pathology types and genetic variations between the patients in the two m^5^C modification pattern groups, which suggests that DNA m^5^C methylation modification did not influence clinical and pathologic features of tumors (Supplementary Figure S2B–G).

### Identification and Functional Annotations of m^5^C Genotype Signatures

**FIGURE 4:**
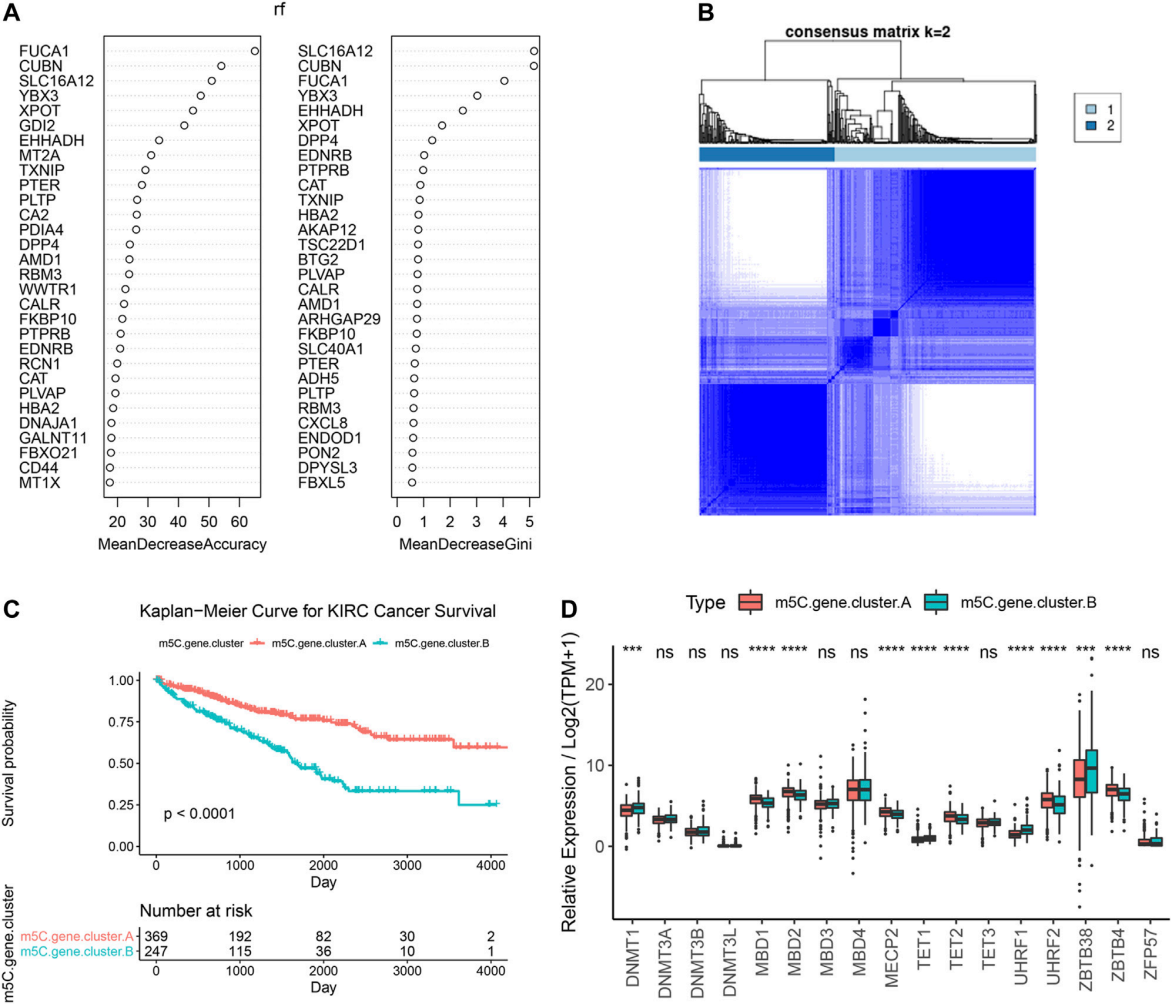
Identification and prognostic implications of m^5^C genotype signatures. **(A)** Random forest results for selecting the most important DEGs. **(B)** Unsupervised clustering based on the expression of selected DEGs. **(C)** Overall survival curves of ccRCC cases in the indicated subgroups. **(D)** Expression levels of 17 m^5^C regulators in the indicated subgroups.

To further explore the biological consequences of the distinct m^5^C modification patterns, we then investigated the genetic constitution of individual m^5^C clusters patterns and identified 180 m^5^C phenotype–related DEGs using the Limma package of R software. Random forest analysis was performed to determine the most important m^5^C gene signatures in identifying m^5^C modification patterns (Figure 4A). To investigate the regulation mechanism of DNA m^5^C modifications on ccRCC, we then performed unsupervised clustering analyses based on the obtained 180 m^5^C phenotype–related signatures to classify patients into different genotypes. Consistent with the clustering of m^5^C modification patterns, the unsupervised clustering algorithm also revealed two distinct m^5^C modification genomic subtypes, named as m^5^C gene clusters A and B (Figure 4B). Kaplan–Meier analysis of ccRCC cases in the combined discovery TCGA and test CPTAC cohorts revealed that patients in the m^5^C gene cluster B group (*n* = 247) showed significantly poor survival compared with cases in m^5^C gene cluster A (*n* = 369) (Figure 4C). Prominent differences in the expression of m^5^C regulators between the distinct m^5^C gene clusters were confirmed using unpaired *t* test, and the results were in accordance with the results of DNA m^5^C methylation modification patterns (Figure 4D). These results revealed the presence of distinct m^5^C methylation modification patterns in ccRCC and showed that these patterns could distinguish aggressiveness in ccRCC.

**FIGURE 5:**
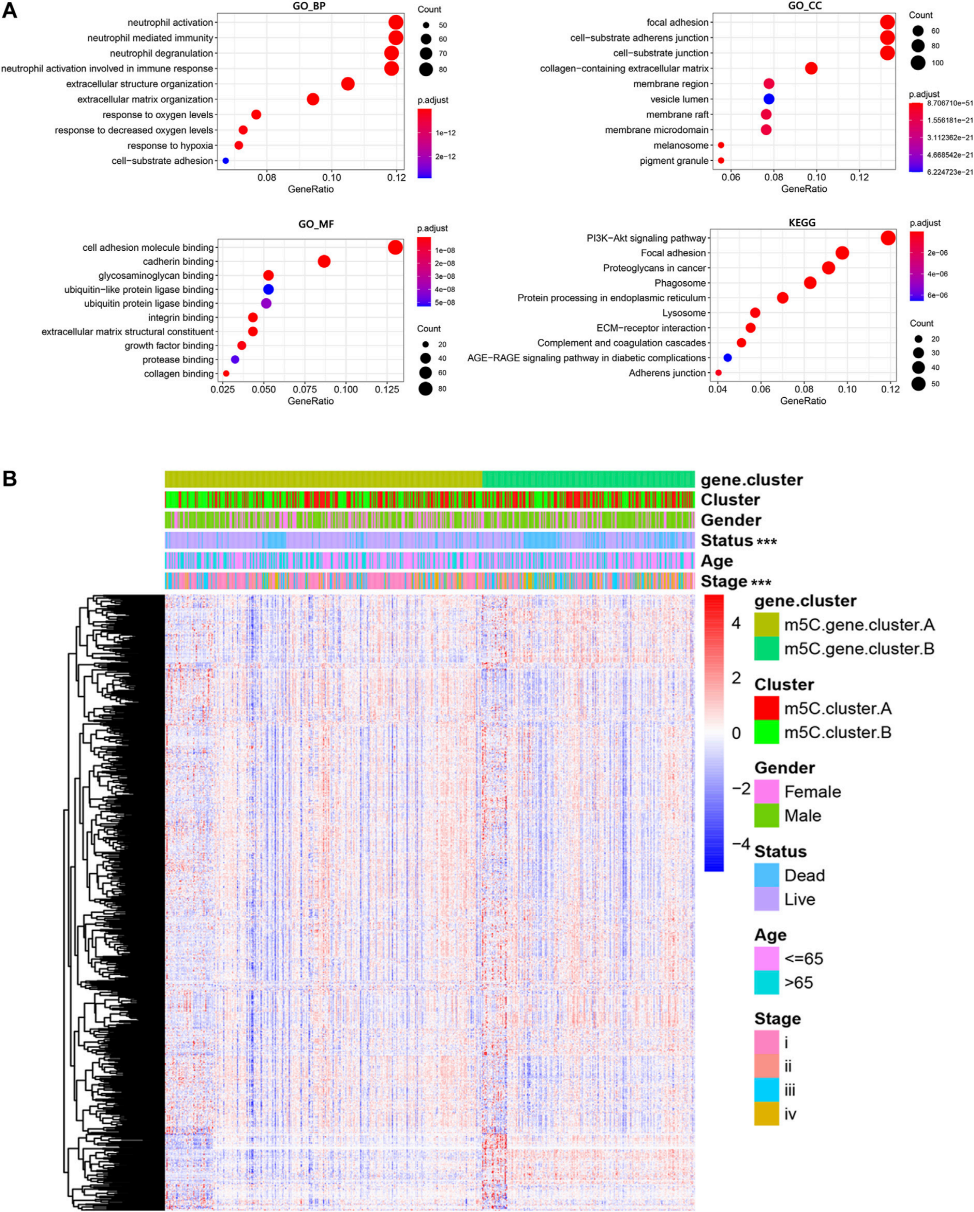
Functional annotations of m^5^C genotype signatures. **(A)** Functional enrichment results of the DEGs. **(B)** Integrative heat map including DEG expression, gender, age, clinical stage, and survival status in the two m^5^C modification pattern groups.

Next, the clusterProfiler package was used to perform GO and KEGG functional enrichment analysis for the m^5^C DEGs. The biological processes, cellular components, and molecular functions with significant enrichment are summarized in Figure 5A. Enriched terms in biological processes were related to m^5^C modification, neutrophil activation–related immune response, and response to hypoxia, which provided a basis that m^5^C modification may play an important role in the immune regulation of the ccRCC TME (Figure 5A). We further found that ccRCC samples in m^5^C gene cluster B showed advanced clinical stages and exhibited higher mortality (Figure 5B). Older patients were concentrated in the m^5^C gene cluster B, and the distinct genotype clusters were characterized by different m^5^C signature genes.

### Generation and Validation of the m^5^C Score Model

**FIGURE 6:**
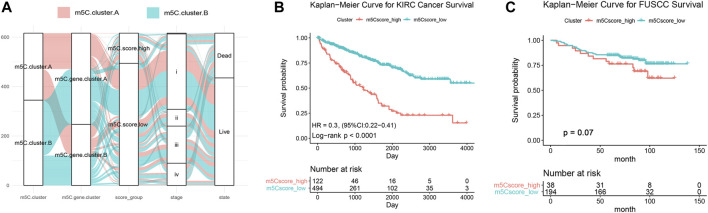
Generation and validation of the m^5^C score model. **(A)** Sanky diagram of the various clusters. **(B)** Overall survival curve of ccRCC patients from TCGA cohort stratified by m^5^C score. **(C)** Overall survival curve of 233 ccRCC patients from FUSCC cohort stratified by m^5^C score.

The above findings demonstrate that the m^5^C methylation modification plays a key regulatory role in reshaping different TME landscapes. Nevertheless, these results were determined on the patient population and might not provide accurate information on survivorship based on m^5^C modification patterns in individual ccRCC patients. Considering the individual intratumor heterogeneity of m^5^C methylation and using the phenotype-related genes, we establish a scoring system for easy quantification of the m^5^C modification patterns for individual ccRCC patients and named this system m^5^C score. The alluvial diagram was applied to visualize the alterations of individual patients (Figure 6A). The m^5^C score clusters prominently classified the patients into two prognostic groups (good and poor) and enabled stratification of patients in both the discovery TCGA and validation real-world FUSCC cohorts. Survival analysis indicated that high m^5^C score was significantly correlated with poor overall survival (HR = 0.3 with 95% CI from 0.22 to 0.41, *p* < .0001) in 516 patients with ccRCC from TCGA (Figure 6B) and correlated with worse overall survival in 266 patients with ccRCC from FUSCC (Figure 6C).

### Relation of m^5^C Modification with Clinicopathological Features and Tumor Somatic Mutation

**FIGURE 7:**
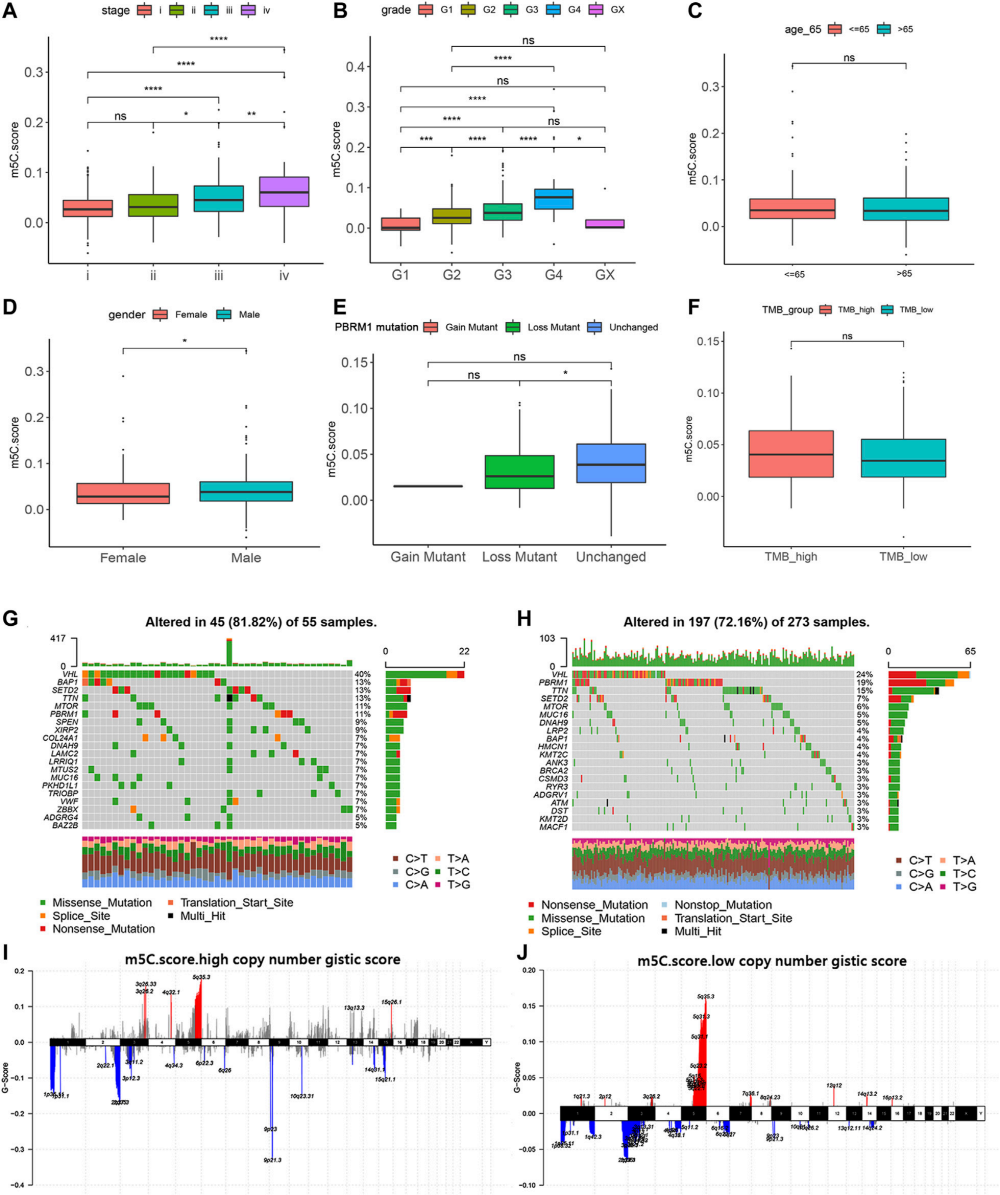
Relation of m^5^C modification with clinicopathological features and tumor somatic mutation. **(A–F)** Associations of m^5^C score with stage, grade, age, gender, *PBRM1* mutation status, and TMB in TCGA cohort. **(G–H)** Landscapes of somatic variants of high and low m^5^C score groups in TCGA cohort. **(I–J)** Copy number variants of high and low m^5^C score groups in TCGA cohort.

We next investigated the relationship of m^5^C score with clinical and pathologic characteristics in ccRCC patients from the training, testing, and validation cohorts. Consistent with its prognostic value, the m^5^C score significantly increased with advancing clinical stages and aggressive ISUP grade and reached the highest level at stage IV or grade 4 (Figures 7A,B). There was no difference in age between the two clusters. The proportion of males in the high m^5^C score group was markedly higher than that of females, which is consistent with the result that male patients have a worse prognosis than female patients with ccRCC (Figures 7C,D).

To reveal the role of the m^5^C score phenotype in the comprehensive molecular landscape of ccRCC, we examined tumor somatic mutation and evaluated DNA variation in the m^5^C score clusters. Patients with mutation in *PBRM1*, a gene frequently mutated in ccRCC, showed a prominently lower m^5^C score compared with patients with wild-type *PBRM1* (Figure 7E). The m^5^C score did not show a significant association with tumor mutation burden in patients with ccRCC (Figure 7F).

We next evaluated the differences in the DNA variation landscape in the two m^5^C score clusters. The top 20 frequently mutated genes in the m^5^C score clusters are shown in Figure 7G,H. *VHL* (mutation frequency, 40%), *BAP1* (13%), *SETD2* (13%), *TTN* (13%), and *MTOR* (11%) were the five most frequently mutated genes in the m^5^C score^high^ group (Figure 7G), whereas *VHL* (24%), *PBRM1* (19%), *TTN* (15%), *SETD2* (7%), and *MTOR* (6%) were the five most frequently mutated genes in the m^5^C score^low^ group (Figure 7H). Thus, we speculate that the significantly higher mutation frequency of *BAP1* in the high m5C score group may contribute to the poor prognosis for ccRCC patients and the low mutation frequency of *PBRM1* may reduce immunotherapy efficiency for ccRCC patients. Copy number variant features are depicted in Figure 7I,J. In addition to the common mutation site located in 5q35.3, copy number variant in m5C score^high^ samples were generally located in 3q25.33, 2q10.53, and 9p12.3 loci.

### Characteristics of TME and Immune Cell Distribution in m^5^C-Related Phenotypes

**FIGURE 8:**
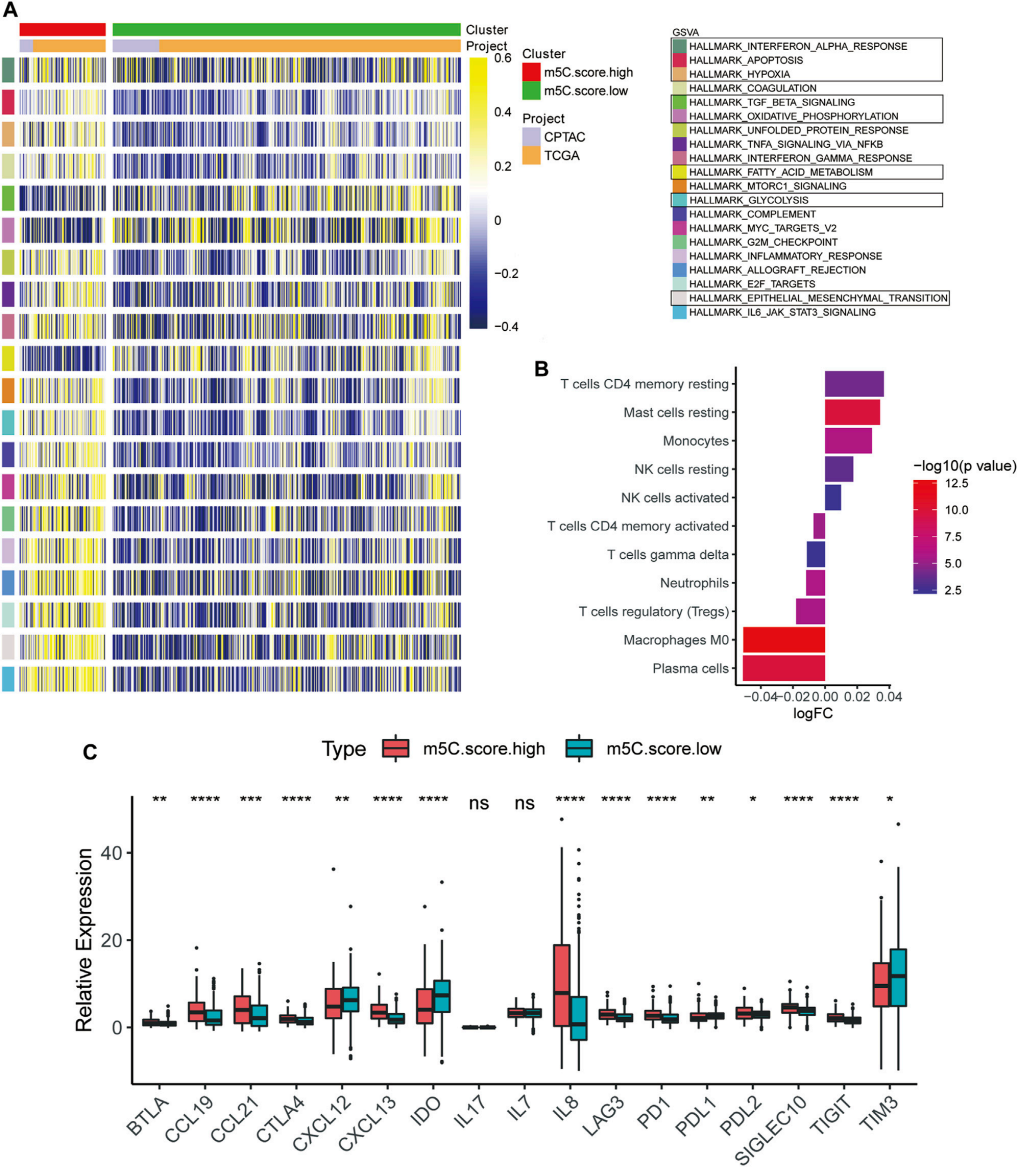
Characteristics of TME and immune cell distribution in m^5^C-related phenotypes. **(A)** GSVA results of high and low m^5^C score groups based on expression profiles from CPTAC and TCGA cohorts. **(B)** Estimation of immune cell infiltration in high and low m^5^C score groups. **(C)** Expression levels of chemokines, cytokines, and immune checkpoints between high and low m^5^C score groups.

To define the role of m^5^C-related phenotypes in regulation of the TME, we first investigated cancer-related pathways characterizing m^5^C gene clusters based on training and testing cohorts. As shown in Figure 8A, TGF-β signaling, oxidative phosphorylation, and fatty acid metabolism were significantly downregulated in ccRCC samples in the m^5^C score^high^ group compared with the m^5^C score^low^ group, whereas pathways involved in protumorigenesis responses of the TME, such as hypoxia, glycolysis, epithelial-mesenchymal translation, and IL6-JAK/STAT3 signaling, were markedly upregulated in the m^5^C score^high^ group. We next evaluated the immune cell infiltration in the TME in m^5^C-related phenotype clusters. The results indicated that CD4^+^ T cell memory resting, mast cell resting, and monocyte and NK cell infiltration significantly correlated with a high m^5^C score, whereas plasma cell, M0 macrophage, Treg cell, and neutrophil infiltration were significantly associated with low m^5^C score in ccRCC patients (Figure 8B). To evaluate the regulatory role of m^5^C score in TME, we explored the expression of chemokine, cytokine, and immune checkpoints in m^5^C score clusters (Figure 8C). We found the expression levels of immune checkpoint factors were significantly different in the m^5^C score^high^ group, suggesting that the high m^5^C score cluster may indicate an immune-suppressive microenvironment.

### Influence of m^5^C Modification Patterns on Chemotherapy and Immunotherapy Response

**FIGURE 9:**
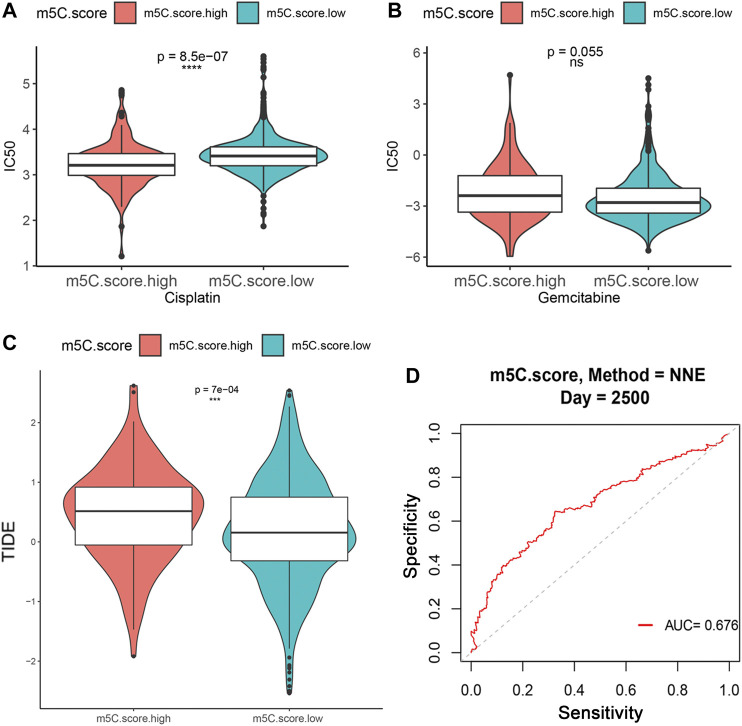
Influence of m^5^C modification patterns on chemotherapy and immunotherapy response. **(A–B)** IC50 value for cisplatin and gemcitabine in low and high m^5^C score groups. **(C)** TIDE prediction score of low and high m^5^C score groups. **(D)** ROC curve for evaluating the ability of m^5^C score to predict immunotherapy response.

Immunotherapies, including anti-immune checkpoints, are revolutionizing the field of cancer therapy. RCC is resistant to traditional cytotoxic chemotherapy but can be responsive to immunotherapy. Therefore, we investigated whether the m^5^C modification signature could predict the responses to chemotherapy and ICTs in the combined ccRCC cohorts (*n* = 860, TCGA, CPTAC, and FUSCC). Evaluation of the ICC50 of cisplatin showed that the low m^5^C score group was significantly correlated with a higher IC50 value, which indicates that the low m^5^C score group may be less sensitive to cisplatin (Figure 9A). However, no significant differences were observed in predicting IC50 values of gemcitabine between the m^5^C modification groups (Figure 9B). The TIDE algorithm was used to predict intratumoral heterogeneity and responsiveness to immunotherapy. The findings indicate that a higher m^5^C score was significantly correlated with an elevated TIDE score, suggesting that the high m^5^C score group may show a reduced response to immunotherapy, such as PD-1 and PD-L1 blockade (Figure 9C). The ROC curve showed a relatively stable ability for predicting the immunotherapy response of m^5^C score with an AUC of 0.676 (Figure 9D).

### TME Characterization in the m^5^C Modification Phenotypes

**FIGURE 10:**
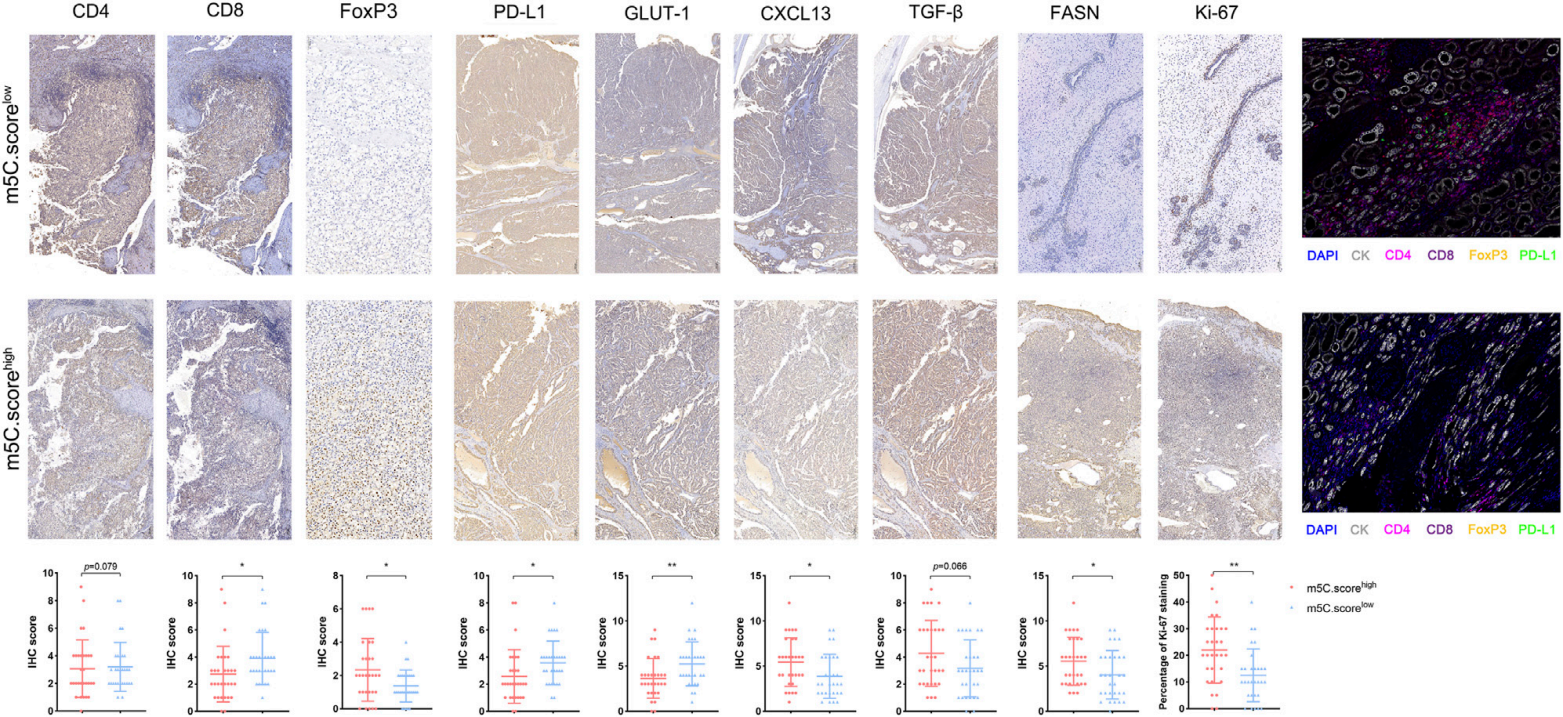
TME characterization in the m^5^C modification phenotypes. TME characterization assessment between high and low m^5^C score groups based on IHC staining (CD4, CD8, FoxP3, PD-L1, GLUT-1, CXCL13, TGF-β, FASN, Ki-67) and opal multimarkers IHC staining (DAPI, CK, CD4, CD8, FoxP3, PD-L1).

To further test the stability of m^5^C score model, we applied the m^5^C score signature established in the real-world FUSCC proteomics cohort and evaluated TME characteristics by IHC staining analysis of 30 consecutive ccRCC tissue sections. IHC staining revealed significantly decreased CD8, PD-L1, and GLUT-1 expression and elevated FoxP3, CXCL13, and FASN expression and Ki-67 staining in tumors from the FUSCC cohort (*p* < .05) in the m^5^C score^high^ group (Figure 10), suggesting immune-suppressive characteristics of the TME. Furthermore, we found a significantly decreased number of infiltrated CD4^+^ T cells and CD8^+^FoxP3^+^ Treg cells and downregulated PD-L1 expression in the immune-cold m^5^C score^high^ group using opal multimarker IHC staining (Figure 10). In general, the data from multiomics bioinformatics to the real world demonstrate that lower m^5^C score predicts better responses to immunotherapy for ccRCC patients.

## Discussion

Increasing evidence demonstrates that malignant biological behaviors of cancer cells are tightly regulated by the TME and genetic variations (Mehdi and Rabbani, 2021). DNA methylation plays an essential role in modulating the transcriptional regulation of genes and subsequent cell functions, including the infiltration and functional differentiation of immune cells participating in protumor and antitumor immune responses (Saleh et al., 2020; Mehdi and Rabbani, 2021; Smiline Girija, 2021). Previous studies mainly focus on tumor-infiltrated lymphocytes or single signatures, and the influence of DNA m^5^C epigenetic regulators on the TME was not comprehensively elucidated. Therefore, the overall characteristics and implications of m^5^C modification patterns on the tumor immune microenvironment in ccRCC warrant further study.

In the current study, we used transcriptome data of 17 DNA methylation regulators and identified two distinct m^5^C methylation modification patterns that are associated with remarkable differences in molecular and clinical characteristics of TME in large-scale ccRCC samples in training, testing, and validation real-world cohorts. The m^5^C score^high^ cluster was characterized by poor prognosis and activation of innate immunity and metabolism, corresponding to the immune-desert phenotype. The m^5^C score^low^ cluster was characterized by the activation of antitumor immunity, corresponding to the immune-excluded phenotype. IHC analysis revealed that the immune-excluded phenotype showed the presence of abundant immune cell infiltrations retained in the parenchyma in ccRCC samples rather than being located in the stroma (Gajewski et al., 2013). This is consistent with our previous findings that, even in occasional cases of nested tertiary lymphatic structures in the immune-excluded phenotype, tumor-infiltrating lymphocytes rarely appear in the stromal component of ccRCC samples (Xu et al., 2021a). Moreover, the immune-desert phenotype, the m^5^C score^high^ cluster, prominently correlates with progressive malignancy, immune tolerance, and lack of T cell–mediated immune responses (Kim and Chen, 2016), guiding effectiveness of immune checkpoint therapy strategies for ccRCC patients.

Research has identified molecular features underlying the initiation and progression of ccRCC. VHL gene inactivation and copy number variation are shown to be involved in promoting the initiation and lethality of ccRCC (D'Avella et al., 2020). The development of sequencing technologies enables determination of the comprehensive DNA mutation landscape and intratumor heterogeneity in the carcinogenesis process (Wettersten et al., 2015; Young et al., 2018; Clark et al., 2019). These findings are extremely important contributions to the categorization and treatment guidance of ccRCC. However, DNA variation, tumor epigenomics, and TME characterizations of ccRCC remain unclear. Here, we find significantly decreased mutation frequency of *VHL* (40% vs. 24%) and *BAP1* (13% vs. 4%) and an elevated mutation frequency of *PBRM1* (11%. vs 19%) in the high m^5^C score cluster compared with the low m^5^C modification pattern. Currently, screening for germline mutations in *BAP1* and *PBRM1* is recommended as these genes may serve as promising targets to predict clinical outcomes and ICT treatment responses (Miao et al., 2018; Gallan et al., 2021; Jonasch et al., 2021). Therefore, we speculate that the significantly higher proportion of *BAP1* mutation in the m5C score^high^ cluster contributes to the poor prognosis for ccRCC patients, and the low proportion of *PBRM1* mutations in the immune-desert phenotype may reflect reduced immunotherapy efficiency of ccRCC patients.

DNA methylation has an important impact on tumor initiation and progression because of its critical role in transcriptional regulation (Bates, 2020). An overall decrease in methylated CpG content is typically observed in tumors, and this leads to genome instability and oncogene activation. CpG hypermethylation in the promoter region of specific genes is a hallmark of many tumors (Paz et al., 2003; Bai et al., 2021). DNA methylations have been identified in genes involved in immune modulation, inflammation, cell differentiation, and metabolic and development processes (Serena et al., 2020). Here, we show that m^5^C methylation modification patterns may function to reshape different metabolism processes and the immune TME landscape, and our results suggest that m^5^C modification may mediate the therapeutic efficacy of ICTs. The m^5^C score together with integrated signatures, including tumor mutation load, PD-L1 expression, T cell infiltration, and immune TME based on multiomics large-scale samples data, may represent an effective predictive treatment strategy. In clinical practice, the m^5^C score can be used to comprehensively assess the m^5^C methylation modification patterns as well as distinct immune cell infiltration of the TME within individuals, allowing for determination of the genetic landscape and immunophenotypes and effective clinical treatment of ccRCC.

## Conclusion

In summary, this work reveals the general regulation mechanisms of DNA m^5^C methylation modification patterns on the tumor immune microenvironment. The m^5^C modification patterns have marked influences on intratumoral heterogeneity and the complexity of the individual TME. Comprehensive assessment of tumor m^5^C modification patterns enhances our understanding of TME cell-infiltrating characterizations and helps establish precision immunotherapy strategies for individual ccRCC patients.

## Main Findings

This work reveals the general regulation mechanisms of DNA m^5^C methylation modification patterns on the tumor immune microenvironment. The different m^5^C modification patterns have marked influences on intratumoral heterogeneity and complexity of the individual TME. Comprehensive assessment of tumor m^5^C modification patterns may enhance our understanding of TME cell-infiltrating characterizations and help establish precision immunotherapy strategies for individual ccRCC patients.

## Data Availability

The datasets presented in this study can be found in online repositories. The names of the repository/repositories and accession number(s) can be found in the article/Supplementary Material. The raw proteome data supporting the conclusions of this article will be made available by the authors, without undue reservation.

## Abbreviations

ccRCC, clear cell renal cell carcinoma; CI, confidence interval; CNV, copy numbers variation; CPTAC, Clinical Proteomic Tumor Analysis Consortium; DEGs, differentially expressed genes; FUSCC, Fudan University Shanghai Cancer Center; GSEA, gene set enrichment analysis; GSVA, gene set variation analysis; HE, hematoxylin-eosin; HR, hazard ratio; ICTs, immune checkpoint therapies; ICGC, International Cancer Genome Consortium; IHC, immunohistochemistry; RCC, renal cell carcinoma; ROC, receiver operating characteristic curve; SNP, single nucleotide polypeptides; TCGA, The Cancer Genome Atlas; TME, tumor microenvironment.
