# Supplementary figures and images for: Integrative 5-Methylcytosine Modification Immunologically Reprograms Tumor Microenvironment Characterizations and Phenotypes of Clear Cell Renal Cell Carcinoma
Source: Front Cell Dev Biol. 2021 Dec 8;9:772436. doi: 10.3389/fcell.2021.772436 (PMC8694268; doi:10.3389/fcell.2021.772436)

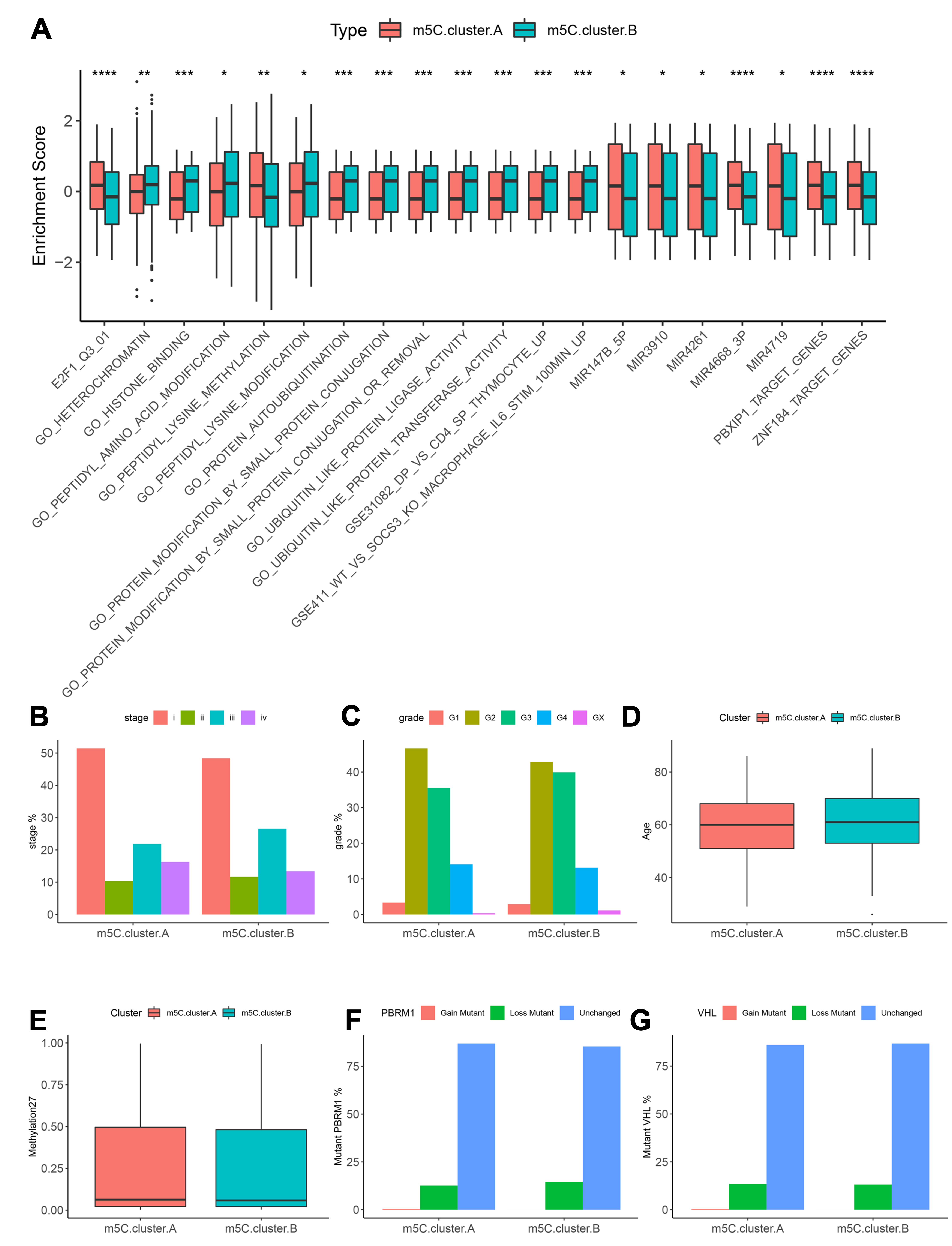

Supplement: Supplementary file 1 [file Image2.TIF]

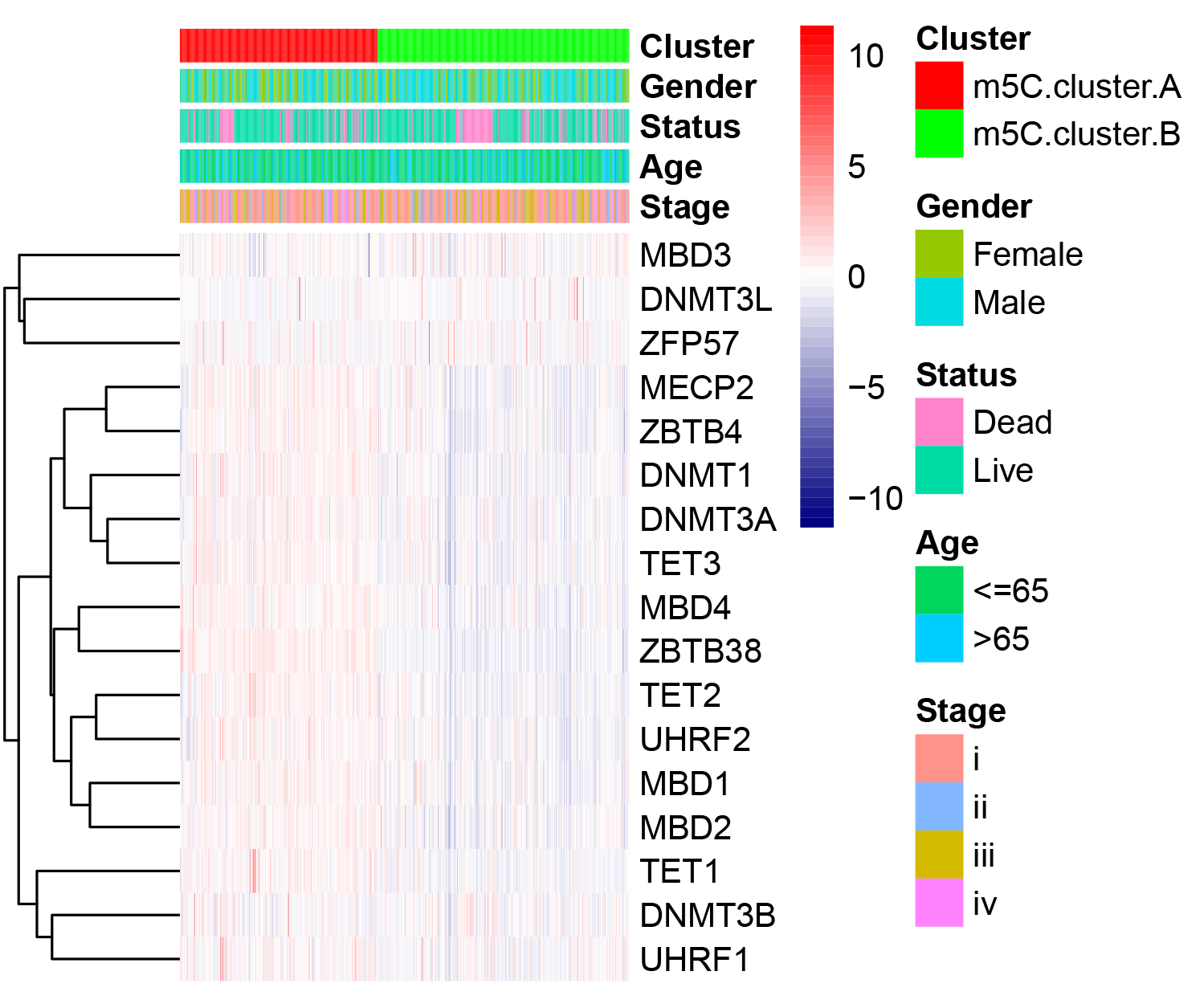

Supplement: Supplementary file 2 [file Image1.TIF]
